# Rapid timescale for an oxic transition during the Great Oxidation Event and the instability of low atmospheric O_2_

**DOI:** 10.1073/pnas.2205618119

**Published:** 2022-09-06

**Authors:** Nicholas F. Wogan, David C. Catling, Kevin J. Zahnle, Mark W. Claire

**Affiliations:** ^a^Department of Earth and Space Sciences, University of Washington, Seattle, WA 98195;; ^b^Virtual Planetary Laboratory, University of Washington, Seattle, WA 98195;; ^c^Space Science Division, NASA Ames Research Center, Moffett Field, CA 94035;; ^d^School of Earth and Environmental Sciences, University of St. Andrews, St. Andrews KY16 9AJ, United Kingdom;; ^e^Blue Marble Space Institute of Science, Seattle, WA 98104

**Keywords:** Great Oxidation Event, photochemistry, oxygen

## Abstract

The Great Oxidation Event (GOE), arguably the most important event to occur on Earth since the origin of life, marks the time when an oxygen-rich atmosphere first appeared. However, it is not known whether the change was abrupt and permanent or fitful and drawn out over tens or hundreds of millions of years. Here, we developed a one-dimensional time-dependent photochemical model to resolve time-dependent behavior of the chemically unstable transitional atmosphere as it responded to changes in biogenic forcing. When forced with step-wise changes in biogenic fluxes, transitions between anoxic and oxic atmospheres take between only 10^2^ and 10^5^ y. Results also suggest that O_2_ between $\sim 10^{-8}$ and $\sim 10^{-4}$ mixing ratio is unstable to plausible atmospheric perturbations. For example, when atmospheres with these O_2_ concentrations experience fractional variations in the surface CH_4_ flux comparable to those caused by modern Milankovich cycling, oxygen fluctuates between anoxic ($\sim 10^{-8}$) and oxic ($\sim 10^{-4}$) mixing ratios. Overall, our simulations are consistent with possible geologic evidence of unstable atmospheric O_2_, after initial oxygenation, which could occasionally collapse from changes in biospheric or volcanic fluxes. Additionally, modeling favors mid-Proterozoic O_2_ exceeding $10^{-4}$ to $10^{-3}$ mixing ratio; otherwise, O_2_ would periodically fall below $10^{-7}$ mixing ratio, which would be inconsistent with post-GOE absence of sulfur isotope mass-independent fractionation.

## Significance

Understanding the rise of atmospheric oxygen on Earth is important for assessing precursors to complex life and for evaluating potential future detections of oxygen on exoplanets as signs of extraterrestrial biospheres. However, it is unclear whether Earth’s initial rise of O_2_ was monotonic or oscillatory, and geologic evidence poorly constrains O_2_ afterward, during the mid-Proterozoic (1.8 billion to 0.8 billion years ago). Here, we used a time-dependent photochemical model to simulate oxygen’s rise and the stability of subsequent O_2_ levels to perturbations in supply and loss. Results show that large oxygen fluctuations are possible during the initial rise of O_2_ and that Mesoproterozoic O_2_ had to exceed 0.01% volume concentration for atmospheric stability.

Abundant atmospheric O_2_ at 21% by volume is the most distinctive and consequential feature of Earth’s atmosphere. Produced by cyanobacteria, algae, and plants, O_2_ is a clear sign of our biosphere that is detectable across interstellar space by telescopic spectroscopy (1). Oxygen permits aerobic respiration, the only known metabolism with sufficient energy yield to sustain complex animal life (2). However, for about the first half of Earth’s 4.5-billion-year-old history, the atmosphere had negligible O_2_ (e.g., ref. 3). This changed ~2.4 billion years ago.

The timing of the Great Oxidation Event (GOE) and the magnitude of atmospheric O_2_ concentrations before and after the GOE can be constrained by the geologic record of sulfur isotopes in combination with photochemical models. Archean and earliest Proterozoic sedimentary minerals contain sulfur isotopes with characteristic mass-independent fractionation (MIF) which abruptly disappears 2.4 billion years ago (4). Sulfur MIF in marine sediments likely requires that atmospheric photochemistry produce elemental sulfur, S_8_ (for explanation, see the introduction in ref. 5) (6, 7). Zahnle et al. (5) used a one-dimensional (1D) photochemical model to show that atmospheric S_8_ production only occurs when atmospheric O_2_ is below $\sim 2\times 10^{-7}$ mixing ratio. An often cited threshold of $2\times 10^{-6}$ was from an earlier photochemical model that did not simulate atmospheres with surface O_2_ mixing ratios between $2\times 10^{-6}$ and $\sim 10^{-15}$ (6). Therefore, the disappearance of the sulfur isotope MIF signal at 2.4 Ga is strong evidence that O_2_ first rose above $2\times 10^{-7}$ mixing ratio then.

Geologic evidence may suggest that the GOE was not a single monotonic rise of oxygen but characterized by oscillations. Using U-Pb dating, Gumsley et al. (8) updated the chronology of sulfur isotope MIF in the stratigraphic record, finding evidence for two oxic-to-anoxic transitions between ~2.4 and ~2.3 Ga. More recently, Poulton et al. (9) report 2.3 Ga to 2.2 Ga marine sediments with sulfur isotopes consistent with approximately five oxic-to-anoxic transitions. Fluctuating O_2_ levels coincide with three to four widespread glaciations, indicating extreme climate instability (10). Overall, geochemical evidence tentatively suggests that O_2_ concentrations and climate were unstable for 200 million years until 2.2 Ga, which marks the most recent estimated timing of the permanent oxygenation of the atmosphere (9). However, interpretations of oscillating O_2_ have been questioned (11). While the geologic evidence for the O_2_ oscillations remains equivocal, the data have raised significant questions regarding the feasibility and timescales for Earth’s great oxidation. Some have argued that the oxygen-rich atmosphere is more stable than an oxygen-poor atmosphere (12), which favors a single rise of O_2_ instead of O_2_ oscillations.

Evidence for O_2_ instability and the time-dependent behavior of O_2_ concentrations has not been reconciled with atmospheric photochemical models. All previous models treated the GOE as successive photochemical steady states (5, 6, 13–19). A photochemical steady state occurs when no atmospheric species changes concentration over time, because their production and loss from reactions and surface sources (e.g., volcanoes or biology) are balanced. Such steady-state calculations have been crucial for understanding the GOE by contextualizing sulfur isotope MIF observations (5, 6), and establishing the relationship between atmospheric O_2_ concentrations and the degree to which O_3_ blocks UV photons from Earth’s surface (i.e., O_3_ shielding) (13, 15, 16), but they do not evaluate time-dependent changes and transient imbalances, or characteristic timescales.

Several theories for the rise of O_2_ suggest that it relied on a global redox titration over 10^8^ y to 10^9^ y involving oxidation of the upper mantle and/or crust, plausibly driven by hydrogen escape, which led to a tipping point where the source flux of O_2_ exceeded a kinetically rapid O_2_ sink from volcanic and metamorphic reductants (20–24). Beyond the tipping point, O_2_ flooded the atmosphere, reaching a new, long-term balance limited by oxidative weathering.

Here, we developed a time-dependent 1D photochemical model capable of investigating changes of O_2_ at the tipping point itself over timescales of 10^2^ y to 10^5^ y rather than the longer-term planetary changes which initiated the GOE. We simulate changing O_2_ as a time-dependent evolution, in contrast to the steady-state approach used in previous studies (e.g., ref. 13), because O_2_ can change on relatively rapid timescales that are not well characterized by steady states. With our model, we compute the time required for an anoxic-to-oxic atmospheric transition, and the time required for deoxygenation. Additionally, we investigate the stability of O_2_ concentrations against perturbations to surface gas fluxes produced by biology. Finally, we use our model results to better constrain O_2_ levels and stability during the GOE (starting at ~2.4 Ga), and during the mid-Proterozoic eon (1.8 Ga to 0.8 Ga).

## Results

**Fig. 1.**
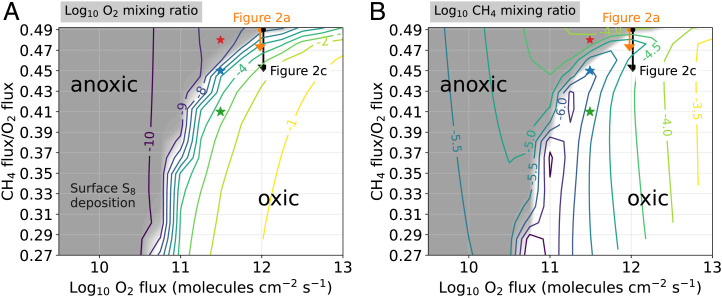
Colored contours show photochemical steady states of (*A*) $\log_{10}$ surface O_2_ mixing ratio and (*B*) $\log_{10}$ surface CH_4_ mixing ratio as a function of $\log_{10}$ O_2_ surface flux and CH_4_ flux/O_2_ flux. Gray shading indicates the magnitude of elemental S_8_ production in the atmosphere, which is considered essential for the preservation of sulfur isotope MIF in marine sediments. Peak S_8_ production is $\sim 10^{7}$ molecules per cm^2^ ⋅s^−1^. Gray shading fades to white for S_8_ production less than $10^{-10}$ molecules per cm^2^ ⋅s^−1^, a negligibly small value. Arrows labeled “Figure 2a” and “Figure 2c” indicate start and end points for time-dependent photochemical models of the oxic transition shown in Fig. 2 *A* and *C*. Red, blue, and green stars are the initial conditions used in the simulations shown in Fig. 4 *B, C*, and *D*, respectively.

To investigate the time-dependent behavior of O_2_ during the GOE, we first computed grids of photochemical steady-state atmospheres. These steady states establish the context for time-dependent photochemical modeling described in subsequent sections. Fig. 1 shows the predicted steady-state surface O_2_ mixing ratio (Fig. 1*A*), the surface CH_4_ mixing ratio (Fig. 1*B*), and the precipitation of atmospheric S_8_ (gray shading) as a function of surface O_2_ flux between $3\times 10^{9}$ and 10^13^ molecules per cm^2^ ⋅s^−1^, and CH_4_ flux/O_2_ flux ratios between 0.27 and 0.49 where gas fluxes are those entering the atmosphere. The surface O_2_ fluxes reported here are net emissions into the atmosphere which exclude recycling within the biosphere. For reference, a comparable model of the modern Earth requires a surface O_2_ flux of 10^12^ molecules per cm^2^ ⋅s^−1^, and a CH_4_ flux/O_2_ flux of 0.09 (CH_4_ flux = $\sim 9\times 10^{10}$ molecules per cm^2^ ⋅s^−1^) (16). We consider a CH_4_ flux/O_2_ flux ratio close to 0.5 to be more realistic for the Late Archean, prior to the GOE, because this ratio is expected if oxygenic photosynthesis is balanced by methanogenesis. In net (21), where “CH_2_O” represents organic matter,

[1] $$\begin{array}{l} {\text{CO}}_{2}+{\text{H}}_{2}\text{O}\to {\text{CH}}_{2}\text{O}+{\text{O}}_{2}\;\;(\text{Oxygenic}\;\text{Photosynthesis})\\ 2{\text{CH}}_{2}\text{O}\to {\text{CH}}_{4}+{\text{CO}}_{2}\;\;(\text{Methanogenesis})\\ {\text{CO}}_{2}+2{\text{H}}_{2}\text{O}\to {\text{CH}}_{4}+2{\text{O}}_{2}\;\;(\text{Net}). \end{array}$$

The CH_4_ flux/O_2_ flux ratio is smaller than 0.5 on modern Earth largely because of the microbial anerobic oxidation of methane via ${\text{SO}}_{4}^{2-}$ in ocean sediments, a process that was unimportant in the anoxic mid-Archean ocean with little sulfate (25–27). We include O_2_ fluxes several orders of magnitude smaller than the modern value ($\sim 10^{12}$ molecules per cm^2^ ⋅s^−1^) because of evidence for smaller primary productivity during the Proterozoic eon (28, 29).

Recall that atmospheric S_8_ deposition is considered necessary to preserve sulfur isotope MIF in ocean sediments (6). We find that S_8_ production is not possible above a $\sim 10^{-7}$ O_2_ mixing ratio (the gray-to-white shading boundary in Fig. 1), consistent with previous results (5).

Fig. 1 uses Archean outgassing surface fluxes for CO, H_2_, H_2_S, and SO_2_ listed in Table 1, with the CO_2_ surface mixing ratio fixed to 1% for all model runs—a reasonable value for the Late Archean according to carbon cycle modeling (30). Additionally, over the same span of surface O_2_ fluxes and H_2_ flux/O_2_ flux ratios, we compute photochemical steady states for the modern fluxes for CO, CH_4_, H_2_S, and SO_2_ listed in Table 1, again fixing CO_2_ to 1%. The results are shown in *SI Appendix*, Fig. S1.

**Table 1.** Fixed surface flux boundary conditions for SO_2_, H_2_S, H_2_, and CO used in this study

Model	SO_2_	H_2_S	H_2_	CO
Archean outgassing*	10^10^	10^9^	$3\times 10^{10}$	$3\times 10^{9}$
Modern values^†^	$3.5\times 10^{9}$	$3.5\times 10^{8}$	$1.22\times 10^{8}$	$2.65\times 10^{11}$

All fluxes have units of molecules per square centimeter per second.

^*^The same fluxes as the “Archean High” values from table 1 in Zahnle et al. (5).

^†^Surface flux values required to reproduce the concentration of each gas in modern Earth’s atmosphere. These values are also the “Case 1” fluxes described in Gregory et al. (16).

In the following sections, we calculate the time required to transition between different steady-state atmospheres shown in Fig. 1 and *SI Appendix*, Fig. S1.

### The Timescale of Atmospheric Oxygenation.

**Fig. 2.**
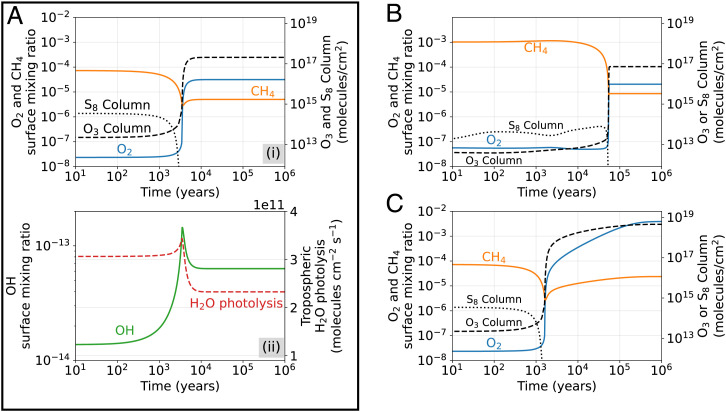
Three models of anoxic-to-oxic transitions. (*A*) Atmospheric oxygenation caused by a step-wise decrease in the methane flux from $4.9\times 10^{11}$ to $4.7\times 10^{11}$ molecules per cm^2^ ⋅s^−1^ (orange arrow in Fig. 1). (*i*) Surface O_2_ and CH_4_ mixing ratios, and O_3_ and S_8_ column abundance over time; (*ii*) OH surface mixing ratio and tropospheric H_2_O photolysis rate. (*B*) Transition caused by step-wise increase in the O_2_ flux from 10^12^ to $1.8\times 10^{12}$ molecules per cm^2^ ⋅s^−1^ and a stepwise increase in the CH_4_ flux to maintain constant CH_4_ flux/O_2_ flux = 0.45 (*SI Appendix*, Fig. S1). Transition in *C* results from a step-wise decrease in the CH_4_ flux from $4.9\times 10^{11}$ to $4.5\times 10^{11}$ molecules per cm^2^ ⋅s^−1^ (black arrow in Fig. 1).

The orange arrow labeled “Figure 2a” in Fig. 1 corresponds to the approximate start and end states of the time-dependent photochemical model run shown in Fig. 2*A*. The model starts with an atmosphere at a steady state, then, at t=0 y, we impose a stepwise decrease in the surface methane flux from $4.9\times 10^{11}$ to $4.7\times 10^{11}$ molecules per cm^2^ ⋅s^−1^ (we keep the surface O_2_ flux constant at 10^12^ molecules per cm^2^ ⋅s^−1^). This perturbation causes O_2_ to rise from $3\times 10^{-8}$ to $3\times 10^{-5}$ mixing ratio over 3,500 y, eliminating photochemical S_8_ production.

The O_2_ transition in Fig. 2 *A*, *i* is modulated by O_3_ shielding of tropospheric H_2_O (13). When a stratospheric O_3_ layer begins to develop, OH production from H_2_O decreases (Fig. 2 *A*, *ii*). Decreasing OH concentrations prevent the mutual annihilation of O_2_ and CH_4_ (by ${\text{CH}}_{4}+\text{OH}\to {\text{CH}}_{3}+{\text{H}}_{2}\text{O}$ followed by ${\text{CH}}_{3}+{\text{O}}_{2}\to \text{products}$), so O_2_ levels increase. The mixing ratio of CH_4_ also rebounds. O_3_ shielding (protecting life on the surface from harmful solar UV radiation) is just barely beginning to operate in this example compared to modern Earth. After the atmosphere reaches a new steady state, the atmospheric column has $3\times 10^{17}$ O_3_ molecules per cm^2^, some 26 times smaller than the modern value of $8\times 10^{18}$ molecules per cm^2^. Note that the extent to which O_3_ shields tropospheric H_2_O can be strongly modulated by 3D dynamical effects (31), which we do not account for.

Like Fig. 2*A*, Fig. 2*B* also shows a transition between a $5\times 10^{-8}$ and $2\times 10^{-5}$ O_2_ mixing ratio, but this model uses the modern outgassing fluxes for CO, H_2_, H_2_S, and SO_2_ listed in Table 1 instead of presumptive Archean outgassing values. Also, at *t* = 0 y, we impose a stepwise increase of the O_2_ flux from 10^12^ to $1.8\times 10^{12}$ molecules per cm^2^ ⋅s^−1^ while keeping the CH_4_ flux/O_2_ flux ratio at 0.45 (see *SI Appendix*, Fig. S1 for context). While the anoxic-to-oxic transition itself still occurs rapidly, the atmosphere simulated in Fig. 2*B* takes 60,000 y to reach the tipping point, which is much longer than the comparable O_2_ transition shown in Fig. 2*A*.

The time required for O_2_ to begin to rise in concentration is controlled by the reservoir of reducing gases, primarily CH_4_, H_2_, and CO, in the preoxygenated atmosphere. Big reservoirs of reducing gases slow the timescale of oxygenation, because reducing gases must be mostly removed before O_2_ can increase. O_2_ cannot increase while reducing gases are abundant, because large oxygen sinks from reactions with reducing gases prevent it. That is why 3,500 y elapse before O_2_ begins to rise in Fig. 2*A*, and why 60,000 y elapse before O_2_ rises in Fig. 2*B*. Fig. 2*B* starts with more reducing gases, which take longer to eradicate.

We can roughly estimate the time required for O_2_ to begin to rise, with a back-of-the-envelope calculation of the rate at which reducing gases are eliminated from the anoxic atmosphere. The total reservoir of reducing gases in the preoxygenated atmosphere in O-equivalent units is

[2] $$N_{\text{reducing}}=\sum_{j}N_{j}\alpha_{j}\approx -4N_{{\text{CH}}_{4}}-N_{\text{CO}}-N_{{\text{H}}_{2}}.$$

Here, $N_{\text{reducing}}$ is the O-equivalent column abundance of reducing gases (O${}_{\text{equiv}}$ molecules per cm^2^) which is equal to the sum of all reducing gases in the atmosphere (*N_j_*) multiplied by *α_j_*, the redox state of each gas. Redox state is a relative quantity that requires defining redox-neutral reference species. Following previous models of early Earth (5), we define H_2_O, SO_2_, CO_2_, and N_2_ as redox neutral, with the oxygen redox parameter $\alpha_{\text{O}}=+1$. Therefore, $\alpha_{\text{H}}=-0.5,\;\alpha_{\text{S}}=-2$, and $\alpha_{\text{C}}=-2$, from redox stoichiometry of hydrogen, sulfur, and carbon, respectively. It then becomes straightforward to calculate the *α_j_* for any molecule. For example, $\alpha_{{\text{CH}}_{4}}=\alpha_{\text{C}}+4\alpha_{\text{H}}=-2-2=-4$. For a more in-depth explanation of atmospheric redox, see section 3 in Harman et al. (32) or chapter 8 in Catling and Kasting (33). $N_{\text{reducing}}$ is approximately equal to the weighted sum of CH_4_, H_2_, and CO because these are the main reducing gases in an Archean Earth-like atmosphere.

The change in column abundance of reducing gases is the difference between the redox columns at the finial and initial atmospheric states.

[3] $$\Delta N_{\text{reducing}}=N_{\text{reducing}}^{\text{final}}-N_{\text{reducing}}^{\text{initial}}.$$

In Fig. 2 *A* and *B*, we initiate the rise of oxygen by changing the surface flux of CH_4_ and/or O_2_ flux. We can quantify this flux perturbation in units of O${}_{\text{equiv}}$ molecules per square centimeter per second ($\Delta F_{{\text{O}}_{\text{equiv}}}$),

[4] $$\begin{array}{ll} \Delta F_{{\text{O}}_{\text{equiv}}} & =\sum_{i}F_{i}^{\text{final}}\alpha_{i}-\sum_{i}F_{i}^{\text{initial}}\alpha_{i}\\ & =(2F_{{\text{O}}_{2}}^{\text{final}}-4F_{{\text{CH}}_{4}}^{\text{final}})-(2F_{{\text{O}}_{2}}^{\text{initial}}-4F_{{\text{CH}}_{4}}^{\text{initial}}). \end{array}$$

Therefore, the time required to oxidize the reducing gases in the atmosphere and permit oxygen to begin rising is approximately

[5] $$\tau_{\text{oxy}}=\left|\frac{\Delta N_{\text{reducing}}}{\Delta F_{{\text{O}}_{\text{equiv}}}}\right|.$$

Plugging in values for the O_2_ transition in Fig. 2*B* yields $\tau_{\text{oxy}}=2,900$ y, a value only slightly smaller than the 3,500 y predicted by the time-dependent photochemical model. For Fig. 2*B*, $\tau_{\text{oxy}}=29,000$ y, which is about a factor of 2 smaller than the figure from 1D photochemistry. Our estimate is too small in this case because the reducing column and its destruction rate are not constant prior to the rise of oxygen (*SI Appendix*). This calculation illustrates that the time required for oxygen to begin rising, once a tipping point of fluxes is reached, mostly depends on the quantity of reducing gases in the preoxygenated atmosphere.

Fig. 2*C* shows a more substantial anoxic-to-oxic transition compared to simulations shown thus far (also see Movie S1). We start with the same steady-state atmosphere as in Fig. 2*A*, except we decrease the methane flux by twice as much at *t* = 0, from $4.9\times 10^{11}$ molecules per cm^2^ ⋅s^−1^ to $4.5\times 10^{11}$ molecules per cm^2^ ⋅s^−1^ instead of $4.7\times 10^{11}$ molecules per cm^2^ ⋅s^−1^ (we keep the surface O_2_ flux constant at 10^12^ molecules per cm^2^ ⋅s^−1^). O_2_ begins to rise and eliminates S_8_ production after ~1,500 y, but O_2_ will reach higher levels because of the lower CH_4_ flux. It takes ~300,000 y for O_2_ to reach its final steady-state abundance of $4\times 10^{-3}$ mixing ratio. While the switch from $10^{-8}$ to $10^{-5}$ O_2_ mixing ratio remains as rapid as in Fig. 2*A*, the predicted increase in O_2_ concentrations to $4\times 10^{-3}$ requires far longer. This timescale is roughly analogous to the time required to deplete H_2_ and CH_4_ reservoirs to allow O_2_ to initially rise in concentration.

In summary, the timescale for O_2_ to rise in concentration depends on the reservoir of redox gases in the atmosphere, and the magnitude of the perturbation to redox surface fluxes. For O_2_ to rise from $10^{-8}$ to $10^{-5}$, reducing gases must first be removed, which can take thousands to 10 thousands of years (Fig. 2 *A* and *B*). Increasing O_2_ concentrations beyond $10^{-5}$ to near percentage levels requires filling a large O_2_ reservoir, which occurs on 10^5^-y timescales in our model run (Fig. 2*C*).

### The Time Required for Deoxygenation.

**Fig. 3.**
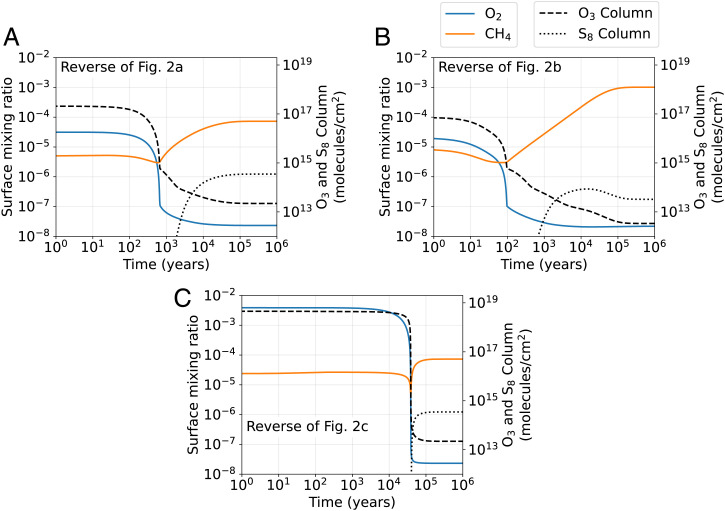
(*A–*C) Simulated reversal of the oxic transitions shown in Fig. 2 *A*, *B*, and *C*, respectively. Each oxic-to-anoxic transition is caused by a stepwise change of the CH_4_ flux and O_2_ flux at *t* = 0 y.

Here, we use our time-dependent photochemical model to address the controversy of the reversibility of the oxic transition (9, 11). Fig. 3 shows the reverse of model runs shown in Fig. 2. For each model run, we start with an atmosphere initially at a steady state at the end of the simulations shown in Fig. 2. Then we impose a stepwise change in the O_2_ and CH_4_ flux at *t* = 0 y to return the atmosphere to anoxia. The reversal of Fig. 2 takes ~700, 100, and 40,000 y, respectively, in comparison to the 3,500, 60,000, and 300,000 y required for oxygenation.

Like the timescale for oxygenation, the timescale of deoxygenation depends on the column abundance of redox-sensitive gases. In the previous section, we established that the timescale required for O_2_ to begin to rise is merely the time required to deplete the reservoirs of CH_4_ and other reducing gases. Analogously, the timescale of deoxygenation is determined by the reservoir of O_2_ and other oxidizing gases in the oxygenated atmosphere. The reversal shown in Fig. 3*B* starts with only $2\times 10^{-5}$ O_2_, which can be depleted very quickly, allowing the return of an anoxic atmosphere. In contrast, the reversal shown in Fig. 3*C* takes 40,000 y because the atmosphere starts with $4\times 10^{-3}$ O_2_, which takes longer to deplete.

### The Stability of Post-GOE Atmospheric Oxygen.

In the previous two sections, we show that reservoirs of redox gases, primarily methane and oxygen, give the atmosphere chemical inertia, controlling the timescale of O_2_ changes. When reservoirs are big, for similar flux perturbations, the O_2_ mixing ratio will change relatively slowly over time; however, when reservoirs are small, photochemistry permits rapid O_2_ transitions. Therefore, the abundance of redox gases in an atmosphere is closely linked to the photochemical stability of oxygen.

**Fig. 4.**
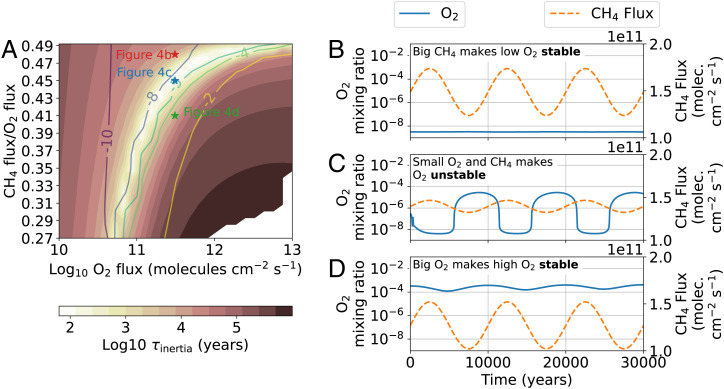
The photochemical stability of O_2_. Shading in *A* shows the steady-state inertial timescale of redox gases (Eq. **6**), and colored contours are the steady-state $\log_{10}$ surface O_2_ mixing ratio (same as Fig. 1*A*). (*B*–*D*) Time-dependent photochemical simulations with oscillating CH_4_ surface fluxes, each beginning with steady-state atmospheres indicated in *A*. O_2_ stability is directly proportional to the column abundance of redox gases in the atmosphere.

Fig. 4*A* shows the steady-state inertial timescale of redox gases, $\tau_{\text{inertia}}$, over the same axes as Fig. 1, which shows mixing ratios. $\tau_{\text{inertia}}$ is the sum of all redox gases in the atmospheric column (O-equivalent molecules per square centimeter), divided by a characteristic flux perturbation, which we take to be 10% of the O_2_ flux,

[6] $$\tau_{\text{inertia}}=\frac{N_{\text{redox}}}{F_{\text{redoxperturb}.}}=\frac{\sum_{i}|\alpha_{i}|N_{i}}{0.1\alpha_{{\text{O}}_{2}}F_{{\text{O}}_{2}}}.$$

We choose the characteristic flux perturbation to be 10% of the O_2_ flux because it is the same order of magnitude as natural redox variations that occur on modern Earth during Milankovitch cycling (see *Discussion*). An upper limit for the characteristic flux perturbation would be 100% of the O_2_ flux. This would decrease all $\tau_{\text{inertia}}$ values in Fig. 4*A* by a factor of 10, which would not change our interpretation. Since CH_4_, CO, H_2_, and O_2_ are the most important redox gases, the numerator in Eq. **6** is well approximated by $4N_{{\text{CH}}_{4}}+N_{\text{CO}}+N_{{\text{H}}_{2}}+2N_{{\text{O}}_{2}}$. Oxygen is the most prone to change for the smallest $\tau_{\text{inertia}}$ values, coinciding with O_2_ mixing ratios between $\sim 10^{-8}$ and $\sim 10^{-5}$ shown in the whitish region of Fig. 4*A*.

The time-dependent photochemical models shown in Fig. 4*B*–*D* illustrate the relationship between $\tau_{\text{inertia}}$ and O_2_ instability. To produce Fig. 4*B*, we started with the steady-state atmosphere indicated on Fig. 4*A*, then imposed 17% amplitude oscillations to the CH_4_ flux with a period of 10,000 y. This forcing had no perceptible effect on the $3\times 10^{-9}$ atmospheric O_2_. A similar 20% CH_4_ flux oscillation also did not significantly perturb an oxic atmosphere starting with $3\times 10^{-4}$ O_2_ (Fig. 4*D*). However, just 5% CH_4_ flux oscillations cause approximately four-orders-of-magnitude oscillations in surface oxygen concentrations for an incipiently oxic atmosphere starting with $3\times 10^{-7}$ O_2_ (Fig. 4*C*). O_2_ is most unstable where the abundance of all redox gases is smallest relative to a characteristic redox surface flux (the whitish area of Fig. 4*A*) between $\sim 10^{-8}$ and $\sim 10^{-5}$ O_2_ mixing ratio. Stability continually increases outside of this range of O_2_ concentrations.

While O_2_ was relatively stable in the Fig. 4 *B* and *D* simulations, it does not mean these atmospheres and initial oxygen concentrations are stable to all atmospheric perturbations. The stability of any O_2_ mixing ratio depends on the atmospheric forcings that are likely in nature. In *Discussion*, we argue that the CH_4_ flux oscillations used in Fig. 4 are realistic because comparable fractional changes in the methane flux have occurred over the past 650,000 y.

Flux oscillations over timescales greater than ~10 y are required to significantly affect O_2_ concentrations. Imposing 100% amplitude fluctuations to the CH_4_ flux with a period of 1 y, starting with the same atmosphere as Fig. 4*C*, did not significantly alter the atmosphere over time. Atmospheres with between $\sim 10^{-8}$ and $\sim 10^{-5}$ O_2_ contain some CH_4_ and O_2_, which gives the atmosphere inertia against annual to decadal change.

## Discussion

Recently, Gregory et al. (16) computed photochemical steady-state atmospheres for a wide range of surface O_2_ and CH_4_ fluxes and found bistable O_2_ concentrations. Their model allows steady-state atmospheres for O_2_ concentrations below $6\times 10^{-7}$ and above $2\times 10^{-3}$ mixing ratio but admits few steady-state solutions in between. They hypothesized that feedbacks between O_2_ and O_3_ shielding eliminate most solutions with these intermediate O_2_ concentrations. In contrast, our model can yield a steady-state solution with intermediate O_2_ concentrations given the right constant surface flux boundary conditions [e.g., Fig. 2*B*; see also Gregory et al.’s (16) figure 8]. The difference might be caused by different steady-state convergence criteria, chemical reaction networks, boundary conditions, or a combination of these factors.

However, a photochemical steady state, for example, at $10^{-6}$ O_2_ mixing ratio, does not mean that such an atmosphere is stable and realistic over 10^5^- to 10^6^-y timescales. Gas fluxes from Earth’s surface can vary during these timescales and significantly change O_2_ concentrations (*Results*).

For example, in the past 650,000 y, the biogenic methane flux has oscillated with an amplitude of 25% ($6\times 10^{9}$ molecules per cm^2^ ⋅s^−1^) and a 100,000-y period (34, 35). On modern Earth, methanogens in wetlands are a major source of atmospheric methane (36). Every 100,000 y, ice sheets have advanced and retreated, covering and uncovering wetlands, changing the CH_4_ flux to the atmosphere. These ice ages and methane flux variations are in response to Milankovich cycles with characteristic periods between 20,000 and 100,000 y. This exact same methane oscillation would not have occurred in the Late Archean or Early Proterozoic because modern wetlands did not exist then, but a similar process involving microbial mats is conceivable.

Zhao et al. (37) modeled cyanobacterial mats on Proterozoic land, finding that they could have been a substantial CH_4_ source to the atmosphere. Ice sheets covering and uncovering microbial mats could have affected global CH_4_ fluxes. Fig. 4*C* illustrates the effect of 5% methane flux variations over Milankovitch timescales on an atmosphere starting with $3\times 10^{-7}$ O_2_. O_2_ oscillates nearly four orders of magnitude between $\sim 10^{-8}$ (anoxic) and $\sim 10^{-4}$ (oxic) (Fig. 4).

An oscillating methane flux is only one of many possible atmospheric perturbations. The Early Proterozoic geologic record preserves evidence of large igneous provinces (LIPs), or massive volcanic eruptions (8). In *SI Appendix*, we show that the H_2_ and CO outgassed from a significant LIP eruption could cause the O_2_ surface mixing ratio to drop from $2\times 10^{-5}$ to $4\times 10^{-9}$ in ~100 y, causing a return to sulfur isotope MIF. In this simulation, we use the maximum LIP eruption rates reported in the literature (38). In addition to LIPs, a Snowball Earth event concurrent with the GOE would have presumably affected gases produced by the biosphere (10).

Constant surface gas fluxes from biology and volcanism for millions of years in the aftermath of the initial rise of O_2_ are unlikely. Additionally, our photochemical modeling shows that, for atmospheres with transitional O_2_ concentrations, relatively small atmospheric perturbations (e.g., a CH_4_ flux change of 5%) over timescales as short as hundreds of years can cause O_2_ to change by orders of magnitude (e.g., Fig. 3*B*). Therefore, substantial variability of O_2_ during the GOE appears possible.

For these reasons, our photochemical modeling results are compatible with recently published evidence of fluctuating sulfur isotope MIF (8, 9) indicating that O_2_ was unstable between 2.4 and 2.2 Ga. We find that shutoff of S_8_ aerosol production, which is required to produce sulfur isotope MIF, occurs at $\sim 10^{-7}$ O_2_ mixing ratio, a region of the parameter space where O_2_ is prone to rapid change (Fig. 4). But, oxygen surface levels between $\sim 10^{-8}$ and $\sim 10^{-4}$ mixing ratio were likely unstable. Short period changes to the biosphere, or volcanic outgassing rates, could have caused order of magnitude O_2_ changes over 100- to 100,000-y timescales. Occasionally, big perturbations to the atmosphere, such as an LIP, might have lowered O_2_ concentrations enough for sulfur isotope MIF to reoccur. Note that the above explanation for the cause of O_2_ oscillations prior to 2.2 Ga is complicated by S-MIF data presented in Izon et al. (11), which do not suggest the same O_2_ variability found by Poulton et al. (9).

After 2.2 Ga, and during the mid-Proterozoic, sulfur isotope MIF never returned. Therefore, this time must have had O_2_ concentrations large enough to prevent O_2_ collapse. Our modeling shows that larger O_2_ concentrations give the atmosphere chemical inertia, slowing atmospheric deoxygenation (Fig. 3). It is therefore challenging to reconcile our modeling results with the interpretation of Planavsky et al. (39), who used Proterozoic chromium isotopes to argue that O_2_ could not have been larger than $2\times 10^{-4}$ mixing ratio. Such a small O_2_ reservoir would have been unstable to LIP eruptions, or variations in the CH_4_ flux from Milankovitch cycles (Fig. 4), which both have evidence of occurring in the mid-Proterozoic stratigraphic record (40–42). We conclude that, for stability, mid-Proterozoic O_2_ levels should have exceeded $\sim 10^{-4}$. This conclusion is compatible with mid-Proterozoic Fe isotopes in ironstones, which suggest O_2_ levels between approximately $2\times 10^{-4}$ and $2\times 10^{-3}$ mixing ratio (43).

Our results are not sensitive to the changing solar UV photon flux between the GOE (~2.4 Ga) and the mid-Proterozoic. Recalculating Fig. 1 using the solar UV flux at 1.3 Ga (44) results in surface O_2_ and CH_4_ surface mixing ratios within a factor of 2 of Fig. 1.

Our work also has implications for the most likely oxygen levels before the GOE. Johnson et al. (45) analyzed molybdenum isotopes in the Archean sedimentary record for signs of continental oxidative weathering. Their work is compatible with two end-member interpretations: 1) If Archean O_2_ was evenly distributed over the globe, then the surface O_2_ mixing ratio was $>3\times 10^{-8}$ and $<2\times 10^{-7}$, or 2) if O_2_ accumulation was geographically restricted, then the O_2_ surface flux was greater than 0.01 Tmol ⋅y^−1^ ($3\times 10^{7}$ molecules per cm^2^ ⋅s^−1^). Our modeling suggests interpretation 1 is unlikely because we find that O_2_ is likely unstable over geologic time for this range of oxygen levels.

In our modeling, we do not explicitly consider redox reservoirs in the oceans, sediments, crust, and mantle, for good reason. These reservoirs are coupled to the atmosphere and can modulate O_2_ levels. However, the timescale of equilibration between the atmosphere and other redox reservoirs is often relatively long (e.g., 100 My for organic carbon in continental sediments), so we consider them to be approximately constant over the timescale of O_2_ transitions ($<10^{5}$ y).

A caveat is that the coupling between redox reservoirs in the atmosphere, crust, or sediments might depend on atmospheric composition. An example is the pyrite oxidation rate, which depends on the partial pressure of oxygen (16). We do not explicitly consider such feedbacks, which could affect the timescale of changing O_2_ levels.

An additional, related caveat is that our model does not consider biological feedbacks. The rise of O_2_ would limit habitats for anaerobes, and permit more widespread aerobic respiration, potentially dropping the CH_4_ flux/O_2_ flux ratio farther than we have modeled here. Also, a stronger ozone UV shield would make new habitats for cyanobacteria and allow the expansion of life on land, promoting chemical and oxidative weathering. All these changes, which we do not explicitly model, would modulate oxygen levels. In this article, we impose changes in the CH_4_ and O_2_ flux that are supposed to be representative of a changing biosphere, but a better model would determine more realistic changes in gas fluxes by directly coupling 1D photochemistry and biology.

## Conclusions

Our time-dependent photochemical modeling of the GOE suggests that oxygen can rise and fall over geologically short periods of time. For an anoxic-to-oxic transition, once a tipping point of imbalanced redox fluxes is reached, the reservoir of reducing gases in the atmosphere must be eliminated before O_2_ can begin to rise. This takes hundreds to 10 thousands of years. O_2_ accumulation to just hundredths or tenths of percent levels requires filling a large O_2_ reservoir, which may occur on a 10^5^-y timescale. Atmospheric deoxygenation occurs over similar periods of time, mainly controlled by the magnitude of the initial O_2_ abundance.

We also find O_2_ instability, especially for mixing ratios between $\sim 10^{-8}$ and $\sim 10^{-5}$. For these O_2_ concentrations, photochemistry demands that both CH_4_ and O_2_ be relatively small in concentration. This small reservoir of redox-sensitive gases permits rapid changes to the atmosphere’s redox state. For example, for an atmosphere starting with $3\times 10^{-7}$ O_2_, 5% amplitude oscillations to the methane flux with a period of 10,000 y cause oxygen to fluctuate four orders of magnitude between anoxic and oxic. Additionally, we show that a LIP eruption could cause the collapse of O_2_ and the return of sulfur isotope MIF for an atmosphere starting with $2\times 10^{-5}$ O_2_ mixing ratio (*SI Appendix*).

We emphasize that the short-term (10^2^ to 10^5^ y) variability in O_2_ levels considered here occurred on the backdrop of the billion-year oxidation of the crust and mantle, and long-term organic burial, which are argued to be the ultimate causes of the rise of oxygen on Earth (e.g., ref. 20).

Overall, our modeling is compatible with, but does not prove, proposed geologic evidence for fluctuating and unstable atmospheric O_2_ after the initial rise of oxygen 2.4 billion years ago. A single, unidirectional, oxidation event remains plausible, although it would require strong and perhaps biological feedbacks promoting permanent substantial changes in the global CH_4_ flux/O_2_ flux ratio. While this is evident between the Archean (CH_4_ flux/O_2_ flux ≈ 0.5) and modern (CH_4_ flux/O_2_ flux ≈ 0.1) biospheres, the dynamics of the Proterozoic biosphere remain largely unexplored. Also, our results suggest that a stable, post-GOE, mid-Proterozoic atmosphere would need an O_2_ mixing ratio exceeding a value in the $10^{-4}$ to $10^{-3}$ range.

## Materials and Methods

To investigate the transition between an anoxic and oxygen-rich Earth, we use a photochemical model with one spatial dimension of altitude, approximating a global average vertical profile. One-dimensional photochemical models are typically governed by a simplification of the continuity equation for molecules,

[7] $$\frac{\partial n_{i}}{\partial t}=-\frac{\partial }{\partial z}\Phi_{i}+P_{i}-L_{i}+R_{\text{i},\;\text{rainout}}+Q_{\text{i},\;\text{lightning}}.$$

Table 2 defines all the variables and their units. Here, the flux ($\Phi_{i}$) is given by

$$\Phi_{i}=-Kn\frac{\partial }{\partial z}\left(\frac{n_{i}}{n}\right)-D_{i}n_{i}\left(\frac{1}{n_{i}}\frac{\partial n_{i}}{\partial z}+\frac{1}{H_{i}}+\frac{1+\alpha_{\text{Ti}}}{T}\frac{\partial T}{\partial z}\right).$$

**Table 2.** Variables in Eq. 7

Variable	Definition	Units
*f _i_*	Mixing ratio of species *i*	Dimensionless
*n_i_*	Number density of species *i*	Molecules per cubic centimeter
*z*	Altitude	Centimeters
*t*	Time	Seconds
*n*	Total number density	Molecules per cubic centimeter
*P_i_*	Total chemical production of species *i*	Molecules per cubic centimeter per second
*L_i_*	Total chemical loss of species *i*	Molecules per cubic centimeter per second
$R_{\text{i,}\;\text{rainout}}$	Production and loss of species *i* from rainout	Molecules per cubic centimeter per second
$Q_{\text{i,}\;\text{lightning}}$	Production and loss of species *i* from lightning	Molecules per cubic centimeter per second
$\Phi_{i}$	Vertical flux of species *i*	Molecules per square centimeter per second
*K*	Eddy diffusion coefficient	Square centimeters per second
*D_i_*	Molecular diffusion coefficient	Square centimeters per second
*H_i_*	$=N_{a}\text{kT/}\mu_{i}g$, The scale heights of species *i*	Centimeters
*H_a_*	$=N_{a}\text{kT/}\bar{\mu }g$, The average scale height.	Centimeters
*N_a_*	Avogadro’s number	Molecules per mole
*k*	Boltzmann’s constant	Ergs per kelvin
*μ*	Molar mass. $\bar{\mu }$ is mean molar mass of the atmosphere, and *μ_i_* is the molar mass of species *i*	Grams per mole
*g*	Gravitational acceleration	Centimeters per square second
$\alpha_{\text{Ti}}$	Thermal diffusion coefficient of species *i*. We neglect this term ($\alpha_{\text{Ti}}=0$)	Dimensionless
*T*	Temperature	Kelvins

The above system of partial differential equations (PDEs) describes how the number density (*n_i_*) of each chemical species *i* changes over altitude and time.

In our photochemical model, we solve a simplified version of Eq. **7** which assumes that the total number density does not change over time (∂n/∂t≈0). This assumption is valid for atmospheric transitions which maintain approximately constant surface pressure and atmospheric temperature. The continuity equations can then be written in terms of mixing ratios (*f_i_*) instead of number densities (see Appendix B.1 in ref. 33 for a derivation),

[8] $$\frac{\partial f_{i}}{\partial t}=-\frac{1}{n}\frac{\partial }{\partial z}\Phi_{i}+\frac{P_{i}}{n}-\frac{L_{i}}{n}-\frac{R_{\text{i},\;\text{rainout}}}{n}+\frac{Q_{\text{i},\;\text{lightning}}}{n}$$

[9] $$\Phi_{i}=-\left(K+D_{i}\right)n\frac{\partial f_{i}}{\partial z}-\zeta_{i}nf_{i}$$

[10] $$\zeta_{i}=D_{i}\left(\frac{1}{H_{i}}-\frac{1}{H_{a}}+\frac{\alpha_{\text{Ti}}}{T}\frac{\partial T}{\partial z}\right).$$

To approximate Eq. **8**, the model replaces the spatial derivatives with finite difference approximations, turning the system of PDEs into a larger system of ordinary differential equations (ODEs). This is the “method of lines” approach to solving a PDE. Catling and Kasting (33), their Appendix B.2, provides a detailed description of how to do this with Eq. **8**; therefore, we will omit a detailed description here, except to point out a sign error. The first two terms for the equation for *B* in their equation B.16 should have minus signs instead of plus signs.

The system of ODEs derived from finite differencing Eq. **8** can be evolved forward in time with numerical integration. However, the photochemical ODEs are “stiff,” meaning that some dependent variables (i.e., the mixing ratios) change much more quickly than others. For example, in the modern atmosphere, OH typically has a chemical lifetime of about 1 s, while CH_4_ has a chemical lifetime of ~10 y. Stiff equations require special, high-stability, “implicit” integration methods. For more details on stiff equations and the implicit methods used to solve them, see ref. 46.

Often, we solve for steady states of the photochemical continuity equation ($\partial f_{i}/\partial t=0$). To find steady states, we begin with some initial atmospheric composition, then integrate Eq. **8** forward in time until the atmosphere ceases to change, that is, a steady state is reached. The assumption of photochemical steady state is approximately valid for most periods of Earth’s history, because the atmosphere changes slowly enough to be in a quasi-steady state.

However, the Paleoproterozoic rise of O_2_ was a relatively fast atmospheric transition that is not well modeled as a photochemical steady-state process. Therefore, describing it requires accurately solving the continuity equation over time.

To model the photochemistry of the GOE, we modified the photochemical model contained within the Atmos modeling suite (described in Appendix B of ref. 33) so that it can accurately solve the time-dependent behavior of Eq. **8**. We call the modified version of the model PhotochemPy. Instead of using a Backward Euler as in Atmos, we used CVODE BDF ODE solver from Sundials Computing (47). CVODE BDF is an implementation of the backward differential formulas (BDF) and is a gold standard for solving large chemical kinetics problems. For details, see *SI Appendix*.

PhotochemPy is open source under a Massachusetts Institute of Technology license. The version of the code (v0.2.14) used in this paper and the corresponding Python scripts to reproduce work done in this article are at https://zenodo.org/record/6824092. However, the most up-to-date version of the code can be found at the following GitHub link: https://github.com/Nicholaswogan/PhotochemPy.

## Supplementary Material

Supplementary File

Supplementary File

## Data Availability

Source code has been deposited in Zenodo (https://zenodo.org/record/6824093#.Ys3KuOzMKCc) (48). PhotochemPy code and the corresponding Python scripts are available at https://zenodo.org/record/6824092. The most up-to-date code is available at https://github.com/Nicholaswogan/PhotochemPy (49).
