# Supplementary material for: Rapid timescale for an oxic transition during the Great Oxidation Event and the instability of low atmospheric O2
Source: Proc Natl Acad Sci U S A. 2022 Sep 6;119(37):e2205618119. doi: 10.1073/pnas.2205618119 (PMC9477391; doi:10.1073/pnas.2205618119)
Supplement: Supplementary File [file pnas.2205618119.sapp.pdf]

PNAS
www.pnas.org

Supplementary Information for

Rapid Timescale for an Oxic Transition During the Great Oxidation Event and the Instability of Low Atmospheric O_2

Nicholas F. Wogan, David C. Catling, Kevin J. Zahnle, and Mark W. Claire

Nicholas Wogan
E-mail: wogan@uw.edu

This PDF file includes:

Supplementary text
Figs. S1 to S4 (not allowed for Brief Reports)
Legend for Movie S1
SI References

Other supplementary materials for this manuscript include the following:

Movie S1

Supporting Information Text

All Great Oxidation Event (GOE) simulations use the solar spectrum at 2.4 Ga, calculated with methods described in Claire et al. (2012) (1). We also always include chemical rainout and NO production from lightning. 95% of steady state simulations conserve redox to a factor better than 10^{-6}. 3% of models, all with $> 0.1\%$ steady-state O_2, conserve redox to a factor of $\sim 10^{-3}$. Figure S1 shows results from steady-state photochemical simulations over the same parameter space as Figure 1 (main text), except using the "Modern Values" fluxes from Table 1 in the main text. Figure S2 shows assumed eddy diffusion and temperature profiles.

Estimating the timescale of the rise of O_2

In the results section of the main text, we estimated the timescale for the rise of O_2 using the following equation:

$$\tau_{\text{oxy}} = \left| \frac{\Delta N_{\text{reducing}}}{\Delta F_{\text{O}_{\text{equiv}}}} \right| \quad [1]$$

Applying this equation to the Figure 2b (main text) simulation:

$$\begin{aligned} \Delta F_{\text{O}_{\text{equiv}}} &= (2F_{\text{O}_2}^{\text{final}} - 4F_{\text{CH}_4}^{\text{final}}) - (2F_{\text{O}_2}^{\text{initial}} - 4F_{\text{CH}_4}^{\text{initial}}) \\ &= (2(1.8 \times 10^{12}) - 4(8.1 \times 10^{11})) - (2(1.0 \times 10^{12}) - 4(4.5 \times 10^{11})) \\ &= 1.6 \times 10^{11} \text{ molecules cm}^{-2}\text{ s}^{-1} \end{aligned} \quad [2]$$

$$\tau_{\text{oxy}} = \left| \frac{\Delta N_{\text{reducing}}}{\Delta F_{\text{O}_{\text{equiv}}}} \right| = \left| \frac{1.47 \times 10^{23}}{1.6 \times 10^{11}} \right| = 9.8 \times 10^{11}\text{ s} = 29,000 \text{ years} \quad [3]$$

This estimation is about a factor of two smaller than the ∼60,000 years predicted by our time-dependent photochemical model.

Figure S3 shows the column of reducing gases and its destruction rate during the Figure 2b simulation. Our estimation for the timescale of oxygenation (Equation Eq. (3)) is off by a factor of two because the reducing column evolves over time, and our estimation of its destruction rate ($\Delta F_{\text{O}_{\text{equiv}}}$) is too large. In the photochemical model, chemistry and transport does not permit a reducing column destruction rates identical to the imposed change in redox fluxes at the surface (1.6×10^{11} O molecules cm^{-2} s^{-1}).

A Large Igneous Province may have destabilized atmospheric oxygen

Here, we show that the H_2 and CO outgassing from large igneous provinces (LIPs), or massive volcanic eruptions, could have potentially caused the collapse of transitional O_2 concentrations ($\sim 10^{-8}$ to $\sim 10^{-4}$ mixing ratio).

To compute H_2 and CO outgassing rates from LIPs, we use the outgassing model described in (2). To briefly summarize, the model estimates the composition of gas bubbles suspended in magma just prior to release into the overlying atmosphere or ocean. Gas composition is computed by solving a system of equations including solubility relationships for H_2O and CO_2, gas-phase equilibrium relationships, and mass conservation of hydrogen and carbon. The model has five inputs: Magma oxygen fugacity, temperature, and overburden pressure, and the H_2O and CO_2 mass fractions ($m_{\text{H}_2\text{O}}^{\text{tot}}$, and $m_{\text{CO}_2}^{\text{tot}}$) in the magma before degassing occurs. For LIPs, we assume a magma oxygen fugacity one log-unit below the fayalite-magnetite-quartz mineral redox buffer (i.e. ΔFMQ-1) following Archean proxies (3). See Chapter 7 in (4) for a description of the fayalite-magnetite-quartz redox buffer. Additionally, we use $m_{\text{H}_2\text{O}}^{\text{tot}} = 0.5$ wt% and $m_{\text{CO}_2}^{\text{tot}} = 0.05$ wt% which agrees with estimates of LIP volatile concentrations (5). Finally, we take the degassing temperature and pressure to be 1473 K and 1 bar, respectfully. With these inputs, our outgassing model predicts 1.6×10^{-2} mol H_2/kg magma and 1.7×10^{-3} mol CO/kg magma.

Converting gas production rates (e.g. mol H_2/kg magma) into gas fluxes to the atmosphere requires estimations of magma eruption rates during LIPs. Basaltic LIPs typically last several million years and cumulatively produce $> 3 \times 10^{18}$ kg magma (6). This magma is release over 10s to 100s of eruptions each lasting several years to 10s of years with eruptions rates between 3×10^{13} to 3×10^{15} kg magma yr^{-1} (7). Multiplying these eruption rates by calculated gas production yields H_2 fluxes between 1.9×10^9 and 1.9×10^{11} molecules cm^{-2} s^{-1} and CO fluxes between 2.0×10^8 and 2.0×10^{10} molecules cm^{-2} s^{-1}.

The time-dependent photochemical simulation shown in Figure S4 shows how atmospheric O_2 responds to maximum LIP H_2 and CO outgassing scenario. The simulation starts with an atmosphere initially at equilibrium, then at $t = 0$ years, we increase the H_2 and CO outgassing fluxes by 1.9×10^{11} and 2.0×10^{10} molecules cm^{-2} s^{-1}, respectfully. O_2 drops from 2×10^{-5} to 4×10^{-9} in 100 years. Basaltic LIP eruptions can last for 10s of years (7), so a 100 year eruption, which is required to cause O_2 to collapse, is within the realm of possibility. However, the period of anoxia would likely be maintained for only a few hundred years, until the eruption ceased. Several periods of anoxia, each corresponding to a significant eruption, might occur during an entire several-million-year LIP event.

It is unclear whether a 100 year period of anoxia is detectable in the geologic record of multiple sulfur isotopes. A single sample from the sedimentary record might represent a period of time greater than 100 years, thus containing sulfur from both an oxic and anoxic atmosphere, diluting the S-MIF signal. Evaluating the detectability of short-term anoxia in the sedimentary record of sulfur isotopes is an interesting target for future work coupling in-situ and bulk rock measurements (8).

LIPs may have additional effects on the atmosphere that we are not accounting for in the above calculations. For example, increased Cl outgassing could catalyze O_3 and CH_4 destruction (e.g. $Cl + O_3 \rightarrow ClO + O_2$ and $Cl + CH_4 \rightarrow HCl + CH_3$), which could have a non-trivial effect on the O_2 concentration.

CVODE numerical integrator

To accurately track atmospheric chemistry over time with our photochemical model, we use the CVODE BDF ODE solver. The reasons CVODE BDF can reliably solve the continuity equation over time are its method of timestep selection and local error control. At each timestep, CVODE estimates the local error that will be introduced with a given timestep size. If the local error is less than the accuracy requirement specified by the user, then the step is accepted and the integration proceeds. Otherwise, the step is rejected, and CVODE retries with a smaller timestep. This general approach is standard in production-grade ODE solvers and will guarantee that each step introduces less local error than that specified by the user (9). The time stepping approach in the older versions of the *Atmos* photochemical model does not estimate the local error introduced with each step, and thus the inaccuracies introduced with integration. This makes it very challenging to determine whether an integration is a good approximation of the true solution or not.

CVODE BDF is also several times faster, depending on the problem, than the backward Euler implementation in older versions of *Atmos*. CVODE uses implementations of the backward differential formulae that are up to 5th order accurate. Backward Euler is 1st order accurate. Higher order ODE integration methods are generally faster because they can take larger timesteps compared to lower order methods while achieving the same accuracy. CVODE also only computes a new Jacobian every few steps when a new one is needed, while the original integrator in *Atmos* computed a Jacobian every single timestep. Jacobians are expensive to compute, thus fewer Jacobian calculations means a faster program.

In addition to implementing time-accurate integration, PhotochemPy is distinct from *Atmos* in a several other ways. The list below highlights the most important differences.

- We restructured the Fortran 77 code using modern Fortran practices. Refactoring in modern Fortran allows more program bugs to be caught during compilation.
- We significantly reduced hard-coding in the model, increasing model generality. PhotochemPy can model vastly different atmospheres such as Saturn, Earth, or Mars with minimal parameter changes which are clear to the user, and not buried deep in the source code.
- We also used the Numpy F2PY tool to generate a deep Python interface to the compiled Fortran (10). This enables easy parallel photochemical integrations using Python multiprocessing tools.
- We introduced extensive call-back errors, which significantly reduces the chances of unnoticed bugs, like a reaction rate which is entered incorrectly.

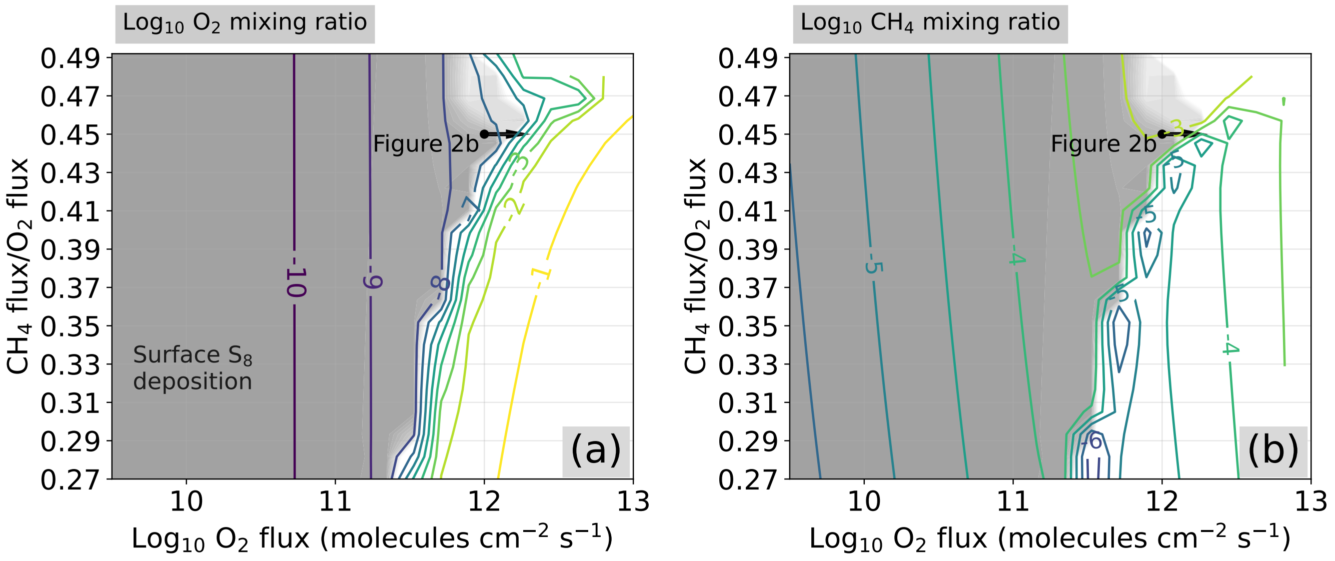

Fig. S1. Identical to Figure 1 in the main text, except we use the "Modern Values" surface fluxes in Table 1 in the main text. The time-dependent photochemical simulation shown in Figure 2b (main text), is indicated with a black arrow.

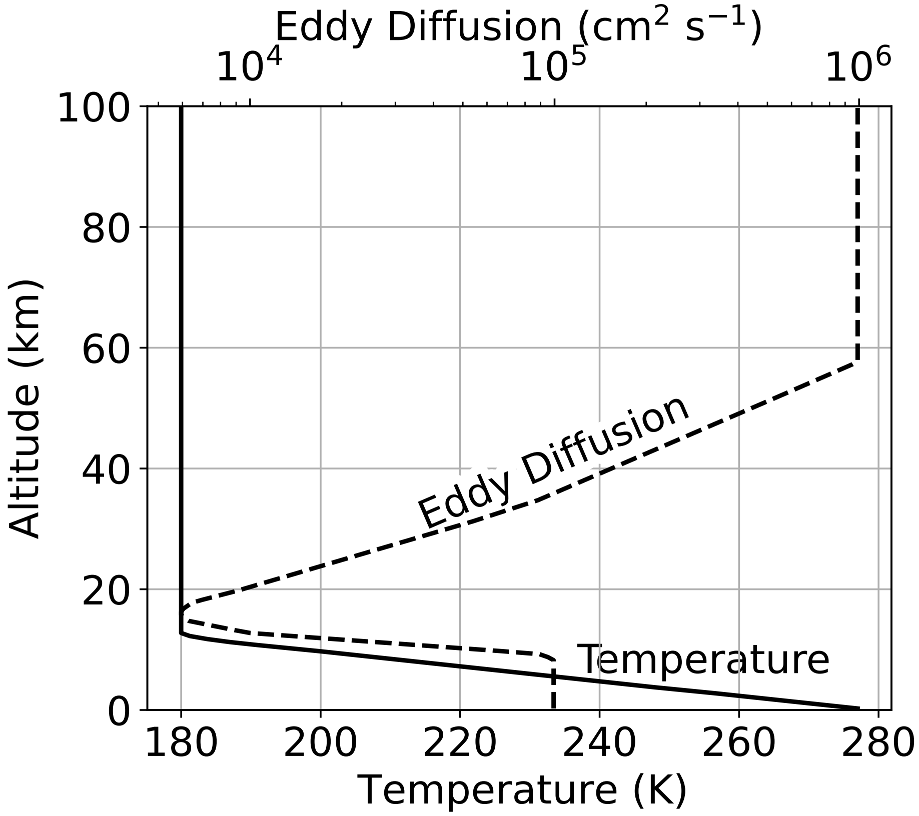

Fig. S2. The temperature and eddy diffusion used for every simulation.

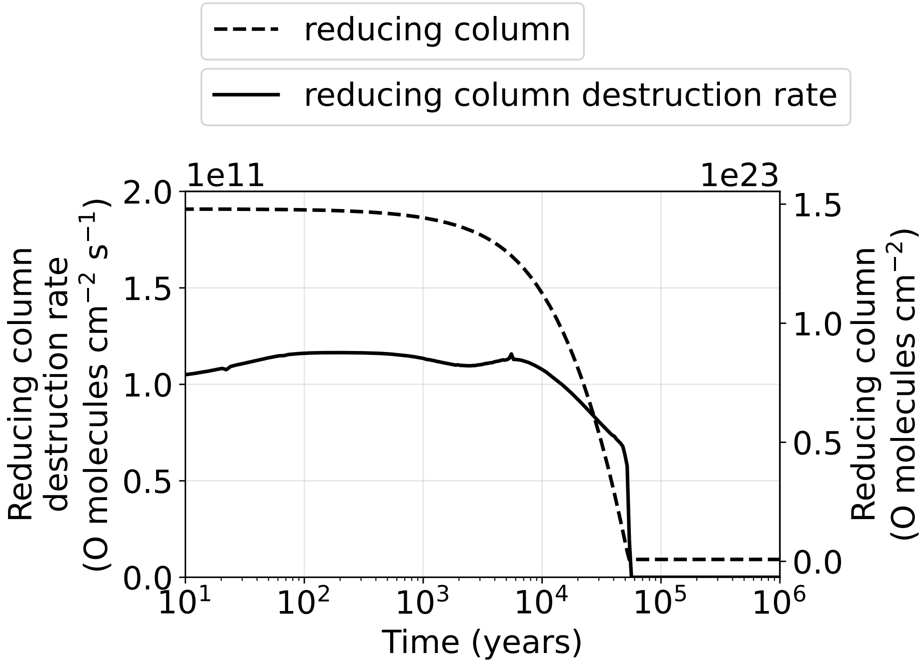

Fig. S3. Reducing gas column and its destruction rate for the simulation shown in Figure 2b.

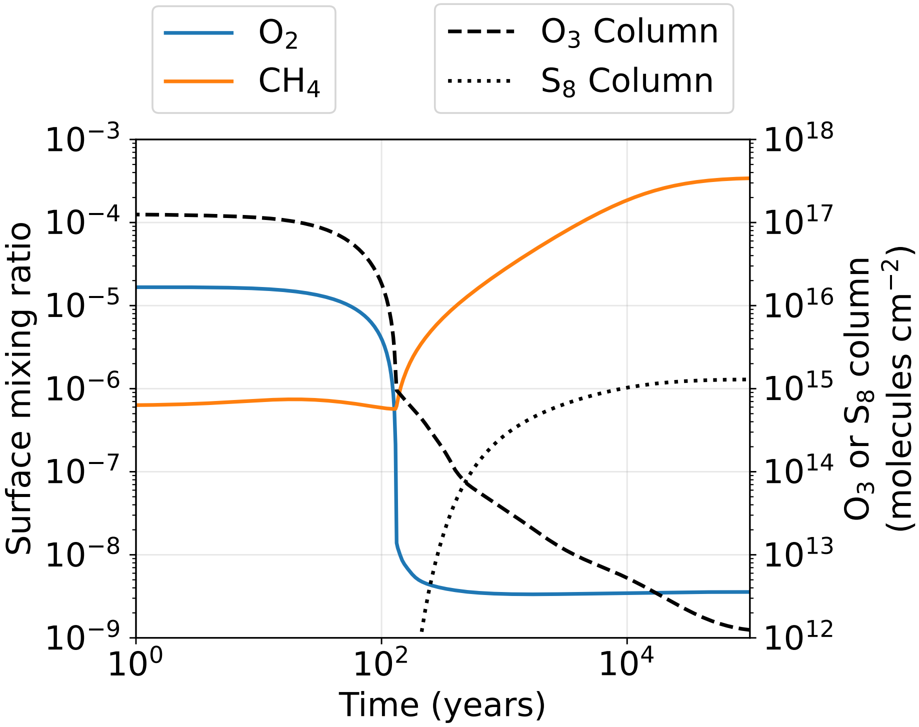

Fig. S4. Modeled oxic to anoxic transition caused by H_2 and CO outgassing from a large igneous province eruption. The simulation begins with a photochemical equilibrium atmosphere, and then is perturbed by a stepwise increase of the H_2 and CO flux by 1.9×10^{11} and 2.0×10^{10} molecules cm^{-2} s^{-1}, respectfully. A LIP eruption should able to produce these outgassing fluxes for the 100 years required to cause O_2 to collapse (see text).

Movie S1. Time dependent photochemical model of the rise of oxygen. This is the same simulation that is shown in Figure 2c in the main text. Left-hand plot shows the mixing ratio of several key species as a function of altitude and time. Right-hand plot shows the photon flux at the top of the atmosphere and the surface as a function of photon wavelength and time. As oxygen rises, and an ozone layer develops, almost all photons < 300 nm are shielded from the surface.

References

1. MW Claire, et al., The evolution of solar flux from 0.1 nm to 160 μm: Quantitative estimates for planetary studies. *The Astrophys. J.* **757**, 95 (2012).
2. N Wogan, J Krissansen-Totton, DC Catling, Abundant atmospheric methane from volcanism on terrestrial planets is unlikely and strengthens the case for methane as a biosignature. *The Planet. Sci. J.* **1**, 58 (2020).
3. S Aulbach, V Stagno, Evidence for a reducing archean ambient mantle and its effects on the carbon cycle. *Geology* **44**, 751–754 (2016).
4. DC Catling, JF Kasting, *Atmospheric evolution on inhabited and lifeless worlds.* (Cambridge University Press), (2017).
5. PJ Wallace, T Plank, M Edmonds, EH Hauri, Volatiles in magmas in *The Encyclopedia of Volcanoes.* (Elsevier), pp. 163–183 (2015).
6. S Self, MF Coffin, MR Rampino, JA Wolff, Large igneous provinces and flood basalt volcanism in *The Encyclopedia of Volcanoes.* (Elsevier), pp. 441–455 (2015).
7. SE Bryan, et al., The largest volcanic eruptions on earth. *Earth-Science Rev.* **102**, 207–229 (2010).
8. N Meyer, A Zerkle, D Fike, Sulphur cycling in a neoarchaean microbial mat. *Geobiology* **15**, 353–365 (2017).
9. E Hairer, G Wanner, *Solving ordinary differential equations II.* (Springer Berlin Heidelberg) Vol. 375, (1996).
10. P Peterson, F2py: a tool for connecting fortran and python programs. *Int. J. Comput. Sci. Eng.* **4**, 296–305 (2009).
